# A Methodological Framework to Capture Neuromuscular Fatigue Mechanisms Under Stress

**DOI:** 10.3389/fnrgo.2021.779069

**Published:** 2021-12-14

**Authors:** Oshin Tyagi, Ranjana K. Mehta

**Affiliations:** Neuroergonomics Lab, Texas A&M University, Industrial and Systems Engineering, College Station, TX, United States

**Keywords:** fatigue, cognition, TMS, EMG, fNIRS (functional near infrared spectroscopy), neuroimaging, central fatigue

## Abstract

Neuromuscular fatigue is exacerbated under stress and is characterized by shorter endurance time, greater perceived effort, lower force steadiness, and higher electromyographic activity. However, the underlying mechanisms of fatigue under stress are not well-understood. This review investigated existing methods of identifying central mechanisms of neuromuscular fatigue and the potential mechanisms of the influence of stress on neuromuscular fatigue. We found that the influence of stress on the activity of the prefrontal cortex, which are also involved in exercise regulation, may contribute to exacerbated fatigue under stress. We also found that the traditional methods involve the synchronized use of transcranial magnetic stimulation, peripheral nerve stimulation, and electromyography to identify the contribution of supraspinal fatigue, through measures such as voluntary activation, motor evoked potential, and silent period. However, these popular techniques are unable to provide information about neural alterations *upstream* of the descending drive that may contribute to supraspinal fatigue development. To address this gap, we propose that functional brain imaging techniques, which provide insights on activation and information flow between brain regions, need to be combined with the traditional measures of measuring central fatigue to fully understand the mechanisms behind the influence of stress on fatigue.

## Problem Statement

In safety critical domains, such as healthcare or emergency response, physical and cognitive stress burden workers strenuously (Benedek et al., 2007; Reichard and Jackson, 2010; Lentz et al., 2019). Workers in these occupations are more frequently exposed to various types of psychological stressors and in some cases, frequent exposure to traumatic events leads to the development of post-traumatic stress disorder (Reynolds and Wagner, 2008; Regehr and LeBlanc, 2017). Due to high physical demand, musculoskeletal injuries like sprains are found to be the most common among emergency responders and accounted for 33–41% of the total reported injuries (Reichard and Jackson, 2010; Orr et al., 2019; Hanson et al., 2021). High physical demands lead to muscle fatigue which can not only significantly impair responder capacity during work—thereby affecting their safety, but can also increase the risk of musculoskeletal disorders in this occupational group (Gallagher and Schall, 2017). In order to develop efficient strategies and techniques to mitigate the harmful effects of psychological and physiological stressors on worker health, the effect of these stressors on their mental and physical health needs to be understood. Several studies have independently investigated the consequences of cognitive stress on the psychological health of individuals and the effect of physical strain on their neuromuscular health and physical performance (Enoka and Duchateau, 2008; Yun, 2016; Gallagher and Schall, 2017; Sawhney et al., 2018). Conventional ergonomics-based models either consider physical factors like posture and muscle activity to assess physical fatigue or use cognitive factors like cognitive stress to assess cognitive fatigue (Matthews, 2002; Ma et al., 2010; Yu et al., 2019). However, the consequences of cognitive stress on neuromuscular health and physical performance have been poorly understood. Cognitive factors and their potential contribution to physical fatigue is often not considered while assessing physical fatigue. Since these two domains, cognitive and physical, are often studied in silos, the interaction between the cognitive and physical domains is not well investigated.

Cognitive stress is a cognitive perturbation that alters executive functioning of the brain responsible for decision making, memory, etc. (Kemeny, 2003; Sandi, 2013). The executive centers of the brain, primarily the prefrontal cortex (PFC), are also responsible for exercise control and exercise termination (Robertson and Marino, 2016). However, no studies so far have investigated how alterations in executive functioning of the brain affect a person's ability to perform exercise and how it influences exercise termination due to fatigue. Understanding the central mechanisms of fatigue under stress is an important first step for the development of interventions that can be used to delay the onset neuromuscular fatigue in safety critical occupations. In this review, the effects of acute stress on neuromuscular fatigue are reviewed, with the focus on central mechanisms of fatigue. First, the mechanisms of neuromuscular fatigue are introduced. The role of central fatigue during physical work and how the brain regulates physical work capacity are also discussed. Building on this body of work, we reviewed studies that provide insights on neural fatigue mechanisms under stress using state of the art neuroergonomics methodologies and present novel physical neuroergonomic methodological framework to capture the neural mechanisms.

## Behavioral Impacts of Stress on Neuromuscular Fatigue

Investigations on the effect of stress on muscle fatigue have mainly been focused on isometric contractions of the upper extremity muscles. These studies have found that cognitive stress adversely affects neuromuscular performance and fatigability (Lundberg et al., 2002; Yoon et al., 2009; Keller-Ross et al., 2014a). For isometric contractions, muscle fatigue, can be defined as a reduction in the ability of muscles to generate force at a required target during exercise (Enoka and Duchateau, 2008). Muscle fatigue is characterized by time to task failure (endurance time), or the exercise induced loss of muscle strength (strength loss). Strength loss can be calculated as the decline in maximum force generating capacity of a muscle after fatiguing exercise relative to their initial force generating capacity. Acute stress applied simultaneously with low intensity contractions is associated with shorter endurance time for contractions of upper extremity muscles but comparable strength loss relative to their initial strength (Yoon et al., 2009; Keller-Ross et al., 2014a; Mehta, 2015). Yoon et al. (2009) found endurance time for sustained elbow flexion was significantly reduced under acute cognitive stress. This reduction was more pronounced in females, where the females exhibited 27.3 ± 20.1% reduction in endurance time under the influence of acute stress, and men exhibited 8.6 ± 23.1% reduction in endurance time. The study also found that the reduction in time to task failure was only observed under high cognitive stress and not under the application of low stressors. Conversely, Mehta and Parasuraman (2014) found no difference in endurance times between the stress and control sessions for sustained handgrip contractions at 30% maximum voluntary contraction (MVC). However, it was not confirmed whether participants were successfully stressed by the mental arithmetic task used to induce high cognitive workload. It is likely that the short time taken to fatigue the muscle for sustained contractions wasn't long enough to induce high level of stress effectively. Mehta (2015) found that stress significantly reduced endurance time and strength loss rate for intermittent handgrip contractions at the same level of 30% MVC and the negative effect of stress was exacerbated by obesity. While stress led to ~18% reduction in endurance time in the non-obese group on an average, this reduction was around 35% in the obese group. These studies suggest that acute stress significantly reduces endurance time and accelerates the onset of fatigue, and that the impact of stress is task-dependent (e.g., sustained vs. intermittent, muscle group tested) and modulated by individual factors (e.g., obesity, gender).

## Mechanisms of Neuromuscular Fatigue

### Peripheral vs. Central Fatigue

Loss of neuromuscular strength is caused by peripheral fatigue produced by changes at or away from the neuromuscular junction and by central fatigue caused by a loss of voluntary activation (defined as the level of neural drive) to the neuromuscular junction (Gandevia, 2001). Peripheral fatigue can develop due to impairments at the neuromuscular junction, changes in the electrical properties of the muscle, development of metabolites interfering with metabolic processes required to maintain Adenosine Triphosphate levels, or the depletion of glycogen available to the muscles (Bigland-Ritchie et al., 1978; Kirkendall, 1990; Amann, 2011). However, the focus of this review is not on peripheral fatigue and therefore, the discussion on peripheral fatigue has been kept brief. Central fatigue is caused due to factors at the supraspinal sites, which control the descending drive to motoneurons or at the spinal sites which form the pathway for the descending drive to reach the neuromuscular junction (Gandevia, 2001; Taylor et al., 2016). Supraspinal factors include changes in the excitability of the motor cortex, or the strength of cortical connections with motoneurons. Spinal factors include intrinsic motoneuron behavior, recurrent inhibition, presynaptic modulation of α- and γ-motoneurons or other influences to the spinal circuitry.

### Central Fatigue During Maximal and Submaximal Contractions

Central fatigue can be defined as an exercise induced loss of voluntary activation. Voluntary activation is the level of neural drive required to activate the muscle received by the neuromuscular junction from the primary motor cortex. This level of neural drive determines the level of force output that will be generated by the muscle. As evidenced by several studies, voluntary activation during exercise is submaximal even during maximal efforts (Gandevia, 2001). This means that even during maximal effort, the neuromuscular system is unable to generate the maximum evocable force that a muscle group is capable of producing. If continuous or intermittent effort is applied over time, voluntary activation further decreases indicating the progression of central fatigue (Todd et al., 2003; Taylor and Gandevia, 2008). During maximal efforts, voluntary activation declines and becomes suboptimal (Bigland-Ritchie et al., 1978; Gandevia et al., 1996) and this decline can be attributed to an increase in recurrent inhibition of the motoneuron pool, reflex inputs from type III and IV muscle afferents (spinal factors) and suboptimal descending drive (supraspinal factors) from the motor cortex (Taylor and Gandevia, 2008). During submaximal contractions, the neural drive to the muscle first increases with time to compensate for peripheral impairments but intermittent maximal efforts with nerve or brain stimulation reveal that superimposed twitch also increases indicating central fatigue (Søgaard et al., 2006). During submaximal contractions the contribution of central fatigue is higher for lower intensity contractions (Ljubisavljević et al., 1996; Eichelberger and Bilodeau, 2007). Supraspinal fatigue has a larger contribution in central fatigue development during submaximal efforts as compared to maximal efforts (Taylor and Gandevia, 2008). This indicates that during submaximal efforts a larger proportion of task failure is caused by a suboptimal descending drive from the motor regions of the brain. This supraspinal fatigue offers scope for improvement in motor performance as fatigue is not caused due to reaching the limits of the neuromuscular system but by a suboptimal drive to begin with.

The rate of recovery of voluntary activation after central fatigue depends on contraction intensity. While, recovery of voluntary activation from maximal or high intensity submaximal contractions takes 2–3 min, recovery from prolonged, low intensity exercise can take up to 60 min (Taylor and Gandevia, 2008). Excitability of the primary motor cortex also reduces with the onset of fatigue and increases during recovery (Benwell et al., 2006; Teo et al., 2012). The changes in cortical inhibition and activation during and after exercise is indicative of the adaptive neural strategies employed by the central nervous system to maintained the drive required for the contracting muscle.

### How Brain Regulates Neuromuscular Performance

To better understand how the descending motor drive is regulated, several models of brain regulation of exercise performance have been proposed. Blain et al. (2016) found the central nervous system inhibits intramuscular metabolic perturbation *via* group III/IV muscle afferents to regulate exercise performance. These results further show that exercise termination due to fatigue is determined by the brain by interpreting the afferent feedback from the muscles. The Central Governor model (CGM) proposes a subconscious “central governor” that regulates the voluntary drive to muscles based on afferent sensory input with the goal of maintaining body homeostasis (Noakes, 2010). According to CGM, physical activity is controlled by a pacing strategy where the “central governor” calculates the intensity of exercise to be maintained and when the exercise would be terminated in order to maintain homeostasis (Gibson and Noakes, 2004). However, according to Marcora (2008), CGM conflicts with conscious effort regulation and does not account for the effect of external motivation on exercise performance. The psychobiological model based on the motivational intensity theory (Wright, 1996) accounts for this conscious control and proposes that task failure occurs when the effort involved in task performance reaches the maximum effort that the participant had calculated to be needed for successful task performance. Although, there is disagreement between the two models on whether exercise regulation is controlled consciously or subconsciously, both models suggest that exercise is regulated by the brain to a calculated safe exertion limit.

As brain regulates effort during exercise, perturbation to certain cortical regions during exercise can also potentially lead to the development of central fatigue before the neuromuscular system reaches capacity. Such perturbations could be concurrent mental fatigue, chronic conditions, or even acute cognitive stress. Studies have shown that high mental workload or concurrent mental fatigue during submaximal isometric contractions are accompanied by a reduction in endurance time and the motor unit firing rate (Mehta and Agnew, 2012; Kowalski and Anita, 2020) and also by a stunted prefrontal cortex (PFC) (Shortz et al., 2015; Mehta, 2016). Studies have shown that changes in PFC activation are associated with changes in motor regions and muscle oxygenation during fatigue development (Rupp and Perrey, 2008; Rupp et al., 2013). However, these studies have not been able to conclusively establish a causal relationship between PFC activation, activation of the motor regions, and neuromuscular fatigue.

## Methods for Capturing Neural Mechanisms of Fatigue

Further review of existing techniques to identify the central mechanisms of fatigue was done in order to understand the capabilities and limitations of these techniques to identify neural mechanisms of fatigue under stress. Table 1 shows the studies that were reviewed to obtain a comprehensive understanding of different bio instruments and techniques used in understanding the mechanisms of fatigue. Nine studies that specifically aimed to identify mechanisms behind fatigue were found. To get a comprehensive understanding of the current methods used to identify influencing factors of fatigue, the methodology, instrumentation and relationship of measurements with fatigue was documented.

**Table 1:** Studies investigating the mechanisms of fatigue.

**S. no**.	**Muscle and contraction type**	**Instrumentation**	**Measurements and their behavior with fatigue**
1	Brief 3 s MVCs of plantar flexion for testing. Fatigue achieved by repeated electrical nerve stimulation to the plantar flexors	Force transducer, surface EMG of the soleus and tibialis anterior muscles, nerve stimulation, cortical activity of M1, S1, and PFC	1. MVC torque↓ 2. rms EMG↓ 3. Area under the curve for HBO for S1↓, M1↓ and PFC 4. Area under the curve for HBR for S1↑, M1↑ and PFC
2	Intermittent maximal voluntary abduction of the index finger with duty cycle 70% for 10 min	Force transducer, EMG from the FDI and ADM muscles, motor nerve stimulation to the ulnar nerve, TMS over the motor cortex.	1. MVC torque↓ 2. M_max_ 3. MEP amplitude in FDI↓ and ADM↓ 4. SICI↓
3	Sustained isometric index finger abduction at 45, 60, and 75% MVC. Brief 5 s MVC before and after exercise for testing	Force transducer, percutaneous stimulation to the FDI	1. MVC torque↓ 2. Time to task failure (↓ with MVC level) 3. VA↓ (only for 30% MVC)
4	Sustained isometric elbow flexion with rms EMG maintained at initial level of at 25% MVC for 10 min, Brief MVCs	Force transducer, EMG of the biceps brachii, stimulation of the Brachial plexus, TMS	1. rms EMG 2. MEP (measured as % M_max_) 3. M_max_ 4. SICI↓ 5. ICF↓
5	Sustained (225 s) and intermittent (duty cycle of 67%; 960 s) isometric handgrip contractions at 30% MVC	Force transducer, surface EMG of the handgrip flexor muscles, fMRI	**Sustained Contractions:** 1. EMG (averaged over 25 s) 2. Force (averaged over 25 s) 3. Brain activation for M1↑, S1↑, PFC↑, SMA↑, CG↑, and CBL **Intermittent Contractions:** 1. EMG (averaged over 25 s) 2. Force (averaged over 25 s)↓ 3. Brain activation for M1↑, S1↑, PFC↑, SMA CG and CBL
6	Maximal isometric elbow flexion sustained for 3 min and brief MVCs	Force transducer, percutaneous stimulation of biceps brachii, TMS over the left M1 and sphygmomanometer cuff to maintain muscle fatigue by ischemia	1. MVC force↓ 2. VA↓ **Recovery with ischemia** 1. MVC force (did not recover) 2. VA (did not recover)
7	Sustained submaximal isometric elbow flexion at 15% MVC for 43 min with brief MVCs in between (every 3 min)	Force transducer, motor nerve stimulation to the brachial plexus, TMS over the motor cortex, and surface EMG at biceps, triceps, and brachioradialis	1. VA from TMS↓ and nerve stimulation↓ 2. RPE↑ 3. M_max_ 4. MEP area↑ 5. Silent period length↑ 6. rms EMG↑
8	Intermittent maximal isometric elbow flexion with duty cycle 50, 60, 75, and 85.7%	Force transducer, TMS over the motor cortex, and surface EMG at biceps and brachioradialis	1. MVC torque↓ 2. TMS evoked twitch↑ 3. MEP amplitude↑ 4. Silent period length↑
9	Sustained maximal isometric elbow flexion for fatigue and brief submaximal flexion at 90, 75, 50, and 25% MVC	Force transducer, motor nerve stimulation to the brachial plexus, TMS over the motor cortex, and surface EMG at biceps, and triceps	1. VA from TMS (↑with contraction intensity) and nerve stimulation (↑with contraction intensity) 2. Superimposed twitch from TMS↑ and nerve stimulation↑ (for maximal efforts) 3. M_max_ 4. MEP amplitude from biceps (large) and triceps (negligible)

*To make the results comparable, only studies on healthy populations were included. The references are as follows: 1 (Alexandre et al., 2015), 2 (Benwell et al., 2006), 3 (Eichelberger and Bilodeau, 2007), 4 (Hunter et al., 2016), 5 (Liu et al., 2003), 6 (Gandevia et al., 1996), 7 (Søgaard et al., 2006), 8 (Taylor et al., 2000), 9 (Todd et al., 2003). The abbreviations used in the table are—abductor digiti minimi (ADM), cerebellum (CBL), cingulate gyrus (CG), deoxy-hemoglobin (HBR), first dorsal interosseous (FDI), motor cortex (M1), oxy-hemoglobin (HBO), prefrontal cortex (PFC), rating of perceived effort (RPE), root mean square EMG (rms EMG), somatosensory cortex (S1) and Voluntary Activation (VA). ↓ denotes “reduced” and ↓ denotes “increased.”*

**Figure 1:**
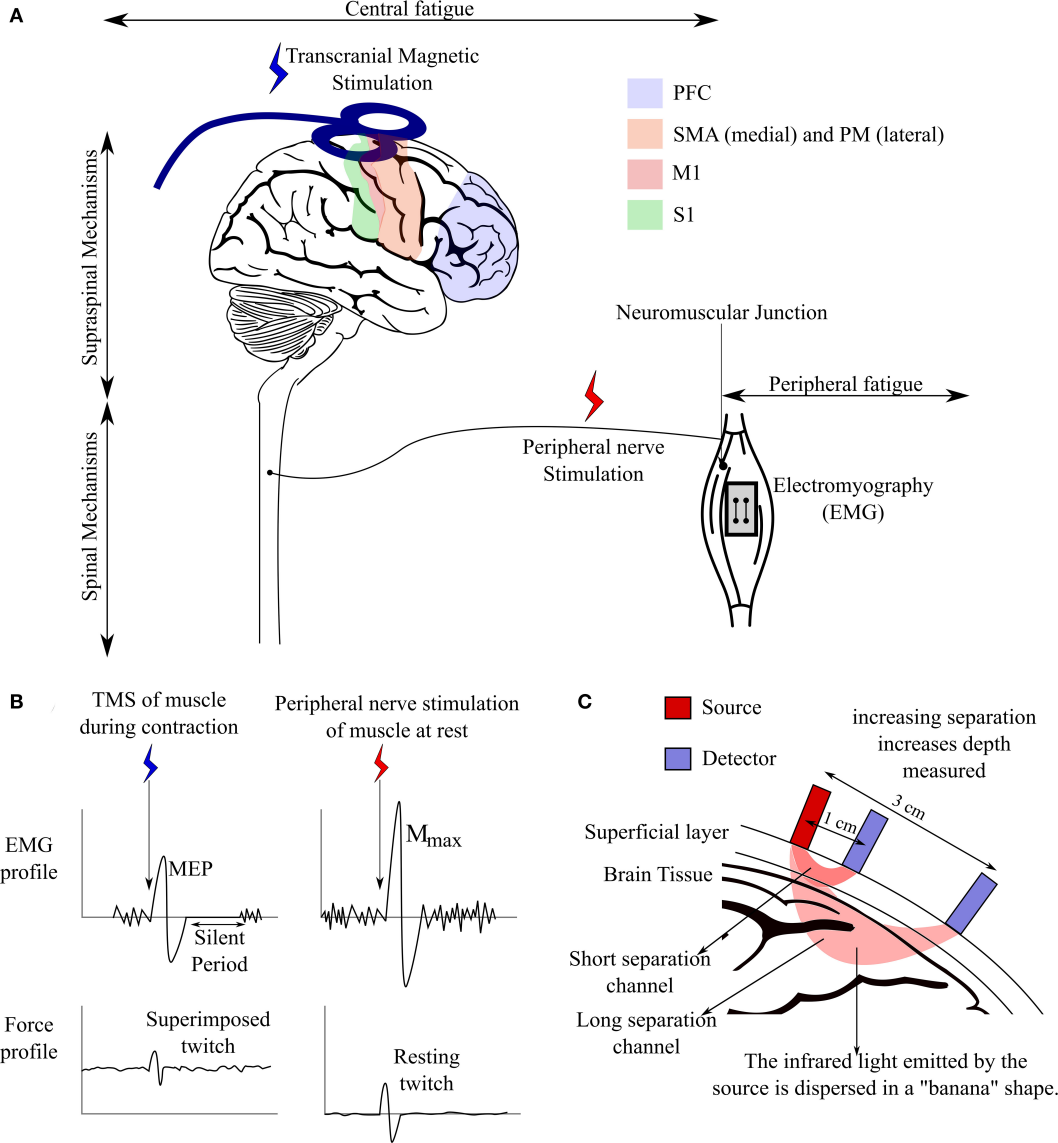
Mechanisms of neuromuscular fatigue and methods identifying these mechanisms. **(A)** Different mechanisms of fatigue acting along the neuromuscular pathway and location of bio instruments used for detecting mechanisms of fatigue involved. Activation and functional connectivity of the superficial cortical regions involved in neuromuscular performance are measured using fNIRS. **(B)** EMG and force profile of muscle during stimulation *via* TMS or peripheral nerve stimulation. **(C)** Brain imaging using fNIRS. Hemodynamic activity in a region is recorded by emitting infrared light of a given intensity in the region and detecting the intensity of light emitted back. The change in intensity is converted to concentration of oxygenated/deoxygenated blood using the Beer–lambert law.

After, the basic techniques and metrics of calculating fatigue were identified, a more in-depth review of these techniques and related metrics was performed. Based on the review, following techniques were identified and illustrated in Figure 1.

### Transcranial Magnetic Stimulation and Peripheral Nerve Stimulation

TMS is a cortical stimulation technique that uses high intensity transient magnetic field to induce an electrical field in the cortical regions (Carroll et al., 2001). Supramaximal stimulation of the motor cortex or the peripheral motor nerve is used to measure voluntary activation by measuring the “twitch” generated by the muscles while performing a voluntary contraction (Todd et al., 2003). The twitch generated by the supramaximal stimulation of the motor cortex or motor nerve represents the extra force the human was not able to generate voluntarily. Traditionally, voluntary activation is calculated using the following formula:


$$\begin{array}{l} \text{Voluntary Activation}=100\times \left(1-\frac{\text{Superimposed twitch}}{\text{Resting Twitch}}\right) \end{array}$$

As discussed earlier, the superimposed twitch can be measured through cortical or nerve stimulation during a voluntary contraction (Gandevia, 2001). In case of nerve stimulation, the resting twitch is measured by the stimulation of muscles at rest, however the same technique cannot be used for cortical stimulation as the excitability of the motor cortical and corticospinal neurons is far less when the muscle is relaxed (Todd et al., 2016). Todd et al. (2003) developed a method of estimation of the resting twitch by calculating the *y*-intercept of a linear regression of the amplitude of the superimposed twitch against the contraction levels from 50 to 100% of maximum voluntary contractions. Therefore, twitch interpolation technique required twitch to be given at different levels of effort above 50% MVC to have enough data for linear regression.

Twitch interpolation by nerve stimulation has been traditionally used in measuring changes in voluntary activation but is limited in interpretation of causal mechanisms that may be behind these changes (Todd et al., 2016). Measuring voluntary activation *via* nerve stimulation can tell us whether the voluntary drive to the neuromuscular junction is submaximal (Allen et al., 1995) and this submaximal drive could be due to inability of motoneurons to discharge continuously, or a diminished excitatory drive to the motoneurons. This does not indicate whether the submaximal drive is due to a suboptimal descending drive from the motor cortex or due to inhibitions in the corticospinal pathways. Voluntary activation obtained from the stimulation of the motor cortex *via* TMS, however, reveals different information than nerve stimulation. If superimposed twitch is generated by motor cortical stimulation, the cortical output was not maximal and not sufficient to drive motoneurons maximally (Taylor et al., 2006). Thus, voluntary activation calculated from cortical stimulation by the TMS represents the level of descending drive from the motor cortex and its loss indicates supraspinal fatigue (Gandevia et al., 1996; Taylor et al., 2000, 2006).

While TMS is better suited to answer questions about mechanisms of fatigue as compared to nerve stimulation, there are some challenges associated with it. While nerve stimulation can be confined to activation of only the agonist muscle group, stimulation *via* TMS is less precise in stimulating only the target muscles (Taylor et al., 2006). Thus, the choice of muscle groups should be such that the relative stimulation of the agonist muscles is minimized (Todd et al., 2016). For example, studies have shown that this effect is minimal for isometric contraction for the elbow flexors where the EMG recordings of the triceps show a very small motor evoked potential (MEP) as compared to the biceps (Todd et al., 2003, 2004). This is because elbow flexors have stronger excitatory connections and are twice as strong as compared to the antagonists (Taylor et al., 2006). Therefore, the use of TMS for measuring voluntary activation is feasible for only certain muscle groups. Additionally, there are also methodological challenges with measuring voluntary activation using TMS during recovery from a fatiguing exercise (Dekerle et al., 2019). Traditionally, the resting twitch using TMS is estimated by consecutively measuring superimposed twitch at three intensities of contractions above 50% MVC. However, Dekerle et al. (2019) found that resting twitch is not estimated reliably using this method and resting twitch estimation requires at least nine superimposed twitches for reliable measurement of Voluntary activation. However, the accuracy of this “nine-point” method can also be compromised during recovery. Since, recovery of the muscle from fatigue is quick, the twitches obtained at different contraction intensities will be at different points of recovery. Therefore, the interpretations that can be made from TMS for measuring Voluntary activation during recovery are limited.

### Electromyographic Response to Cortical Stimulation

Surface EMG is often used in studies to monitor neuromuscular fatigue in real time, and analysis of EMG signals gives insight into the physiological mechanisms of fatigue. Studies in the past have shown that surface EMG activity increases linearly with force and that the increase in low frequency signals is characterized by fatigue (Gandevia, 2001). However, surface EMG signals measure compound muscle action potential at the muscle site and do not directly indicate the amount of “neural drive” to the muscles. This is because the muscle action potential (MAP) measured at the site is the result of superimposed MAPs of several muscle fibers and intracellular muscle fiber action potentials. Furthermore, these signals also depend on the conductivity of different muscle fiber types and local blood supply to the site. Therefore, EMG by itself is not a reliable indicator of upstream behavior and is used with nerve or cortical stimulation to measure spinal or supraspinal factors involved in neuromuscular fatigue (Carroll et al., 2001; Hunter et al., 2008; Sundberg et al., 2018). The following features of the EMG response along with cranial or nerve stimulation are used to quantify the mechanisms of fatigue:

1. **M**_max_**:** The electromyographic response obtained from the supramaximal stimulation of the motor nerve associated with the contracting muscle (M_max_) is the maximum potential that can be obtained from the site (Gandevia, 2001). Since M_max_ represents the EMG response to maximal drive to the neuromuscular junction, other EMG responses from cortical stimulation or voluntary contraction are often measured against M_max_ (Todd et al., 2003; Sundberg et al., 2018). Since the size of the evoked potential depends on muscle specific factors and electrode placement, the raw amplitude of each muscle must be normalized to the M_max_ of that muscle to allow for comparison between individuals and before and after fatigue. (Todd et al., 2016). M_max_ along with twitch amplitude can also indicate peripheral fatigue. For their study, Søgaard et al. (2006) found that during submaximal contractions, the resting twitch decreased without any observed changed in M_max_, indicating that there was a decline in force generating capacity of the muscle without any decline in the neural drive.
2. **Motor evoked potential (MEP):** Supramaximal stimulation of the primary motor cortex using TMS produces a short-latency electromyographic response called MEP (Carroll et al., 2001; Todd et al., 2016). MEP is responsible for producing the twitch contraction used in interpolation. As the neural networks activated within the motor cortex include both excitatory and inhibitory axons, MEP represents a balance of both excitatory and inhibitory influences and is less than M_max_ (Carroll et al., 2001). Since motoneuronal excitability increases with activity, the amplitude of MEP increases with contraction intensity (Ugawa et al., 1995). For the agonist muscle directly involved in generating force required to complete a task, the amplitude of MEP must be >50% M_max_ and for the antagonist muscle producing the opposing force to the agonist, the MEP amplitude should be less than 20% M_max_ for optimal twitch (Todd et al., 2016). MEP amplitude remains inhibited even after a non-fatiguing task, and the duration of post-exercise inhibition of MEP is longer for long duration, low intensity contractions as compared to high intensity contractions (Teo et al., 2012). This indicates that, corticomotor excitability post-exercise is reducing and this inhibition is prolonged for long duration, low intensity exercise.
3. **Silent period:** When an EMG response is evoked simultaneously with cortical stimulation, it is immediately followed by a period of no EMG activity known as the silent period. This silent period reportedly lasts for 100–300 ms and the lengthening of the silent period is often interpreted to indicate intracortical inhibition and therefore, supraspinal fatigue (Søgaard et al., 2006; Cogiamanian et al., 2007; Taylor and Gandevia, 2008). The length of the silent period indicates whether the activity of the inhibitory circuits in the motor cortex and the inhibition is mediated by the gamma-Aminobutyric acid (GABA) receptors, particularly, the GABAb receptors (Udupa, 2021). The silent period for the first 50–80 ms is considered to be due to inhibitions at the spinal level (Fuhr et al., 1991; Ziemann et al., 1993), whereas, if the silent period stretches for more than 100 ms, it is considered to reflect cortical inhibition (Søgaard et al., 2006). Yacyshyn et al. (2016) however, found that the influence of spinal factors on the silent period evoked by TMS is much longer than 150 ms. Therefore, the silent period alone cannot be used a measure of supraspinal fatigue and any interpretations and conclusions drawn from the silent period need to be backed by further evidence.
4. **Short interval cortical inhibition:** When a paired pulse TMS paradigm is used with a subthreshold condition stimulus followed by a suprathreshold test stimulus after 1–6 ms, the intracortical inhibitory circuits are activated that result in a stunted MEP. This phenomenon is called Short-Interval Cortical inhibition or SICI (Wagle-Shukla et al., 2009; Hunter et al., 2016). Evidence from present literature supports that SICI is caused by the trans-synaptic activation of the GABA circuits in the primary motor cortex which inhibit or reduce the neuronal excitability in the nervous system (Ziemann et al., 1996; Hanajima et al., 1998; Di Lazzaro et al., 2000). Studies have shown that SICI decreases with the onset of fatigue for both maximal and submaximal voluntary contractions (Benwell et al., 2006; Hunter et al., 2016). This phenomenon is not attributed to central fatigue but is seen as a central adaptation to motor tasks by reducing the inhibition of the motor cortex and ensuring adequate excitability as a response to fatigue. While, SICI is not a direct indicator of muscle fatigue, this metric is useful for understanding the adaptive strategies that might be adopted by the central nervous system to delay the onset of fatigue.
5. **Intracortical facilitation:** Intracortical facilitation is also achieved by a paired pulse TMS paradigm where the interval between the subthreshold stimulus and the suprathreshold test stimulus is 8–30 ms (Wagle-Shukla et al., 2009; Hunter et al., 2016). While, the mechanisms behind intracortical facilitation are not fully understood, Ni et al. (2007) hypothesized that multiple mechanisms both at cortical and spinal levels are likely responsible for this facilitatory effect. Therefore, ICF is not ideal for locating the mechanisms of fatigue as its interpretations are too confounding based on present literature.

While the features above have been extensively studied to understand the mechanisms of fatigue, sex differences in these features have also been studied. Sex differences have been found EMG signals of the lower extremity muscles during fatigue inducing exercise (Cioni et al., 1994; Clark et al., 2005). Pitcher et al. (2003) also found females to have a higher variability in MEP amplitude than males for hand muscles. However, Hunter et al. (2006) did not find sex differences in voluntary activation during exercise. No studies were found to have investigated Sex differences in SICI. Therefore, since there is some evidence of overall sex differences in EMG activity, sex differences for the above EMG features should also be studied.

### Neuroimaging

**Figure 2:**
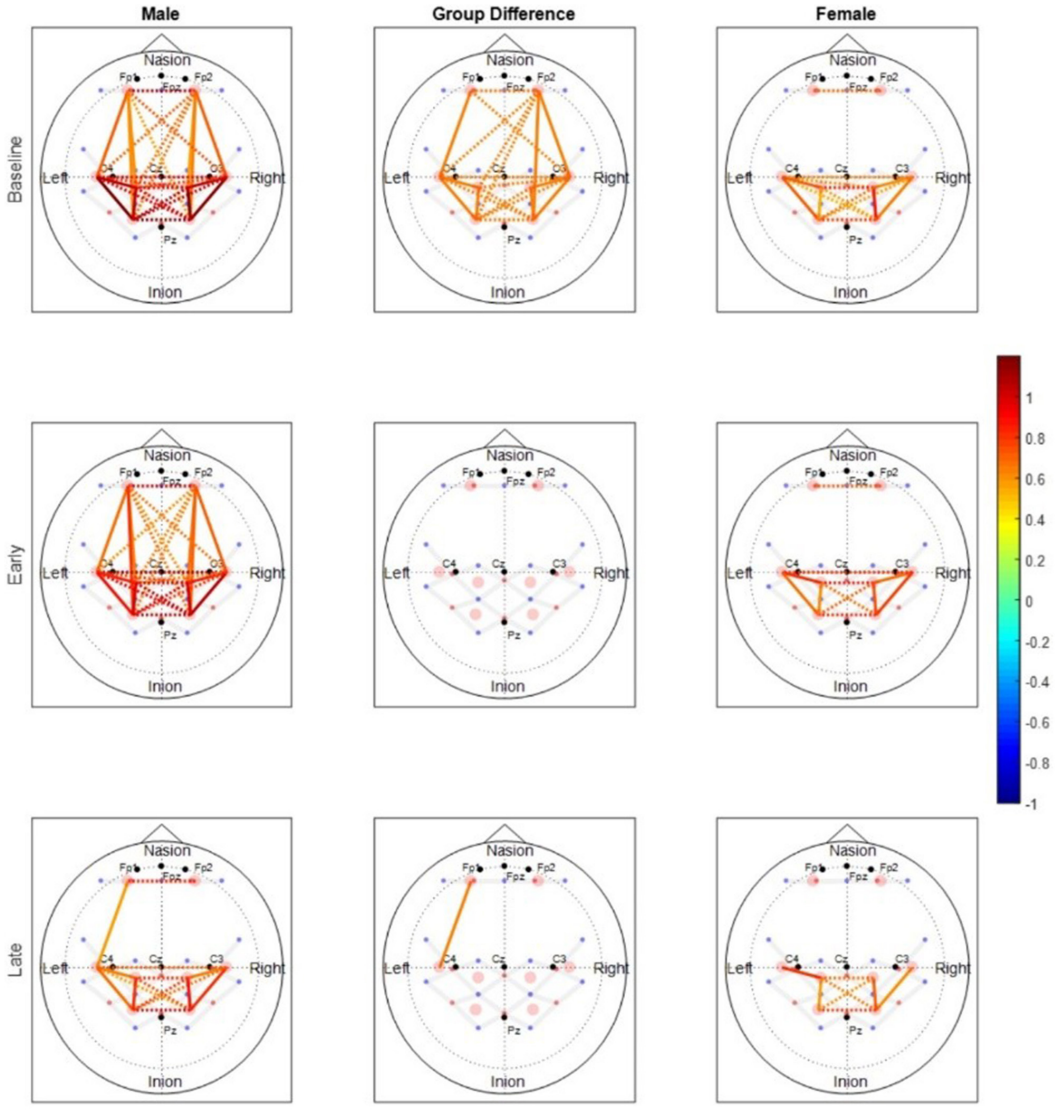
Rhee and Mehta (2018), functional connectivity maps for males and females during submaximal (30% MVC) intermittent handgrip fatiguing protocol. The baseline period involves no physical exercise, the early period is from the early stages of exercise before the onset of fatigue and the late period is after the onset of fatigue during exercise. Color of each node depicts the strength of connectivity between regions. Nodes with solid lines indicate intra-hemispheric connectivity, and nodes with dotted lines indicate inter-hemispheric connectivity. Middle column shows the nodes where connectivity was significantly different between the sexes. The connectivity maps show that the strength of functional connectivity decreases with fatigue.

While the techniques discussed before are efficient in identifying mechanisms of fatigue downstream of the descending voluntary drive from the motor cortex, they are not able to identify mechanisms upstream of this descending drive. Functional brain imaging techniques like electroencephalography (EEG), functional magnetic resonance imaging (fMRI), and functional near-infrared spectroscopy (fNIRS) have made it possible to study brain activity during neuromuscular performance and fatigue manifestation to bridge this gap (Liu et al., 2003; Alexandre et al., 2015; Rhee and Mehta, 2019). Just like EMG provides valuable information about the electrical activity in the muscle while performing a physical task and therefore, indirectly measures the level of neural drive reaching the muscle, brain imaging can capture the activity of the cortical regions involved in sending and regulating the neural drive descending from the primary motor cortex (M1). Brain imaging techniques can monitor cortical activity concurrently with neuromuscular performance which can provide a valuable insight into the central mechanisms of fatigue that TMS cannot provide as it cannot be used concurrently with physical tasks that are at a submaximal level. Of the brain imaging techniques, commonly used in studies on neuromuscular performance, fMRI allows for the greatest spatial resolution and is also capable of measuring cortical regions located deep into the skull (Rao et al., 1993). However, fMRI does not allow for ambulatory examinations or high temporal resolution as the participants need to be supine during fMRI scans (Zhu et al., 2019). While fNIRS is only able to measure hemodynamic activity in cortical regions closest to the brain, it allows for the greatest freedom of movement (Bunce et al., 2006; Zhu et al., 2019). Conversely, while EEG has a better temporal resolution than fNIRS, localization of source of activity is harder for EEG leading to poor spatial resolution (Blinowska and Durka, 2006). However, several source localization algorithms, like LORETA, BPNN, FOCUSS, and more widely used Bayesian techniques, have helped improving the spatial resolution of EEG (Asadzadeh et al., 2020). There are still some methodological challenges with these algorithms with respect to spatial accuracy, and susceptibility to noise. Additionally, EEG is also more susceptible to motion artifacts as compared to fNIRS and requires stronger artifact removal methods (Mehta and Parasuraman, 2013; Zhu et al., 2019). Therefore, taking into account, the advantages of fNIRS over other imaging techniques for functional studies involving movement, further review was conducted on the features that are extracted from hemodynamic activity of the cortical regions (that fNIRS measures). Figure 1C illustrates the method of measuring hemodynamic signal using fNIRS over the brain. The following are the relevant features that can be obtained from fNIRS:

1. **Cortical activation:** fNIRS measures activation in cortical regions by measuring the hemodynamic activity in that region. Studies measuring activation of different cortical regions concurrently with exercise performance and fatigue development have identified M1, the Supplementary and premotor areas (SMA, PM), the somatosensory cortex (S1) and the Prefrontal cortex (PFC) to be involved in motor performance (Liu et al., 2003; Rhee and Mehta, 2018). M1 and S1 are directly involved in the output and control of the descending drive (Liu et al., 2003), where M1 is directly responsible for the motor output and S1 controls the descending drive. SMA and PM are involved in motor planning and the involvement of these motor regions increases as complexity of the tasks increases (Tanji, 1994; Hoshi and Tanji, 2007). Additionally, the premotor areas have also shown to increase activation with increase in cognitive demands suggesting that these areas of the motor system get recruited in conditions to support cognitive functions (Küper et al., 2016; Marvel et al., 2019). The PFC is primarily involved in cognitive control and executive functions (Miller and Cohen, 2001) but PFC activity is also correlated with fatigue development (Rupp and Perrey, 2008; Rupp et al., 2013). As mentioned before, a potential explanation for this correlation is that the PFC co-ordinates with the premotor and other areas of the brain, and uses afferent feedback from the muscles to regulate exercise. Due to their involvement with exercise and/or the influence of cognitive perturbations on their activity, activation of the abovementioned cortical regions during exercise should be monitored to better understand the role of these regions in exercise performance and potential changes under the influence of stress.
2. **Cortical connectivity:** While cortical activation informs how cortical regions behave individually, cortical connectivity measures explain more about the ease with which the cortical regions communicate (Rubinov and Sporns, 2010; Nguyen et al., 2018). Functional connectivity measures the temporal correlation between brain regions and measures how the activity of different brain regions is functionally segregated or integrated (Friston, 1994). Most studies on neuromuscular performance have investigated functional connectivity (Liu et al., 2007; Karim et al., 2017; Rhee and Mehta, 2018). These studies have found that functional connectivity between motor regions decreases with the onset of fatigue (Figure 2). Studies have also found sex differences in functional and structural connectivity of the brain (Ingalhalikar et al., 2014; Rhee and Mehta, 2018). In undirected functional networks however, only the correlation between the activation of two regions is known, and the direction of the information flow between the two regions is not known. Also, it is difficult to determine if the correlated activity in two brain regions is because of mutual influence or due to common inputs from basic functional activity of the brain. These gaps can be bridged by calculating effective connectivity between regions by using techniques like granger causality or directional phase transfer entropy that help in determining the causal influence of one region of the brain on another (Ding et al., 2006; Urquhart et al., 2020).

## Cognitive Stress and Its Effect on the Body

Cognitive stress can be defined as the state when the environmental and task demands are perceived to be beyond individual capacity and there is a perceived threat to individual well-being if the demands are not met (Calvo and Gutierrez-Garcia, 2016). The physiological response to stress leads to the activation of two major systems in the body, the quick autonomic nervous system responsible for the “fight or flight response”, and the slower hypothalamus–pituitary–adrenal (HPA) axis (Vogel and Schwabe, 2016). The HPA activation leads to the release of cortisol from the adrenalin cortex which reaches peak concentration about 20-30 min after stress is induced (Joëls and Baram, 2009). Therefore, cortisol levels are often measured to confirm if stress is induced in an individual (Yoon et al., 2009; Keller-Ross et al., 2014a). Other physiological measures that are also used to measure stress response are skin conductance and heart rate variability (Jacobs et al., 1994; Taelman et al., 2009). The State portion of the State-Trait anxiety inventory (STAI), which is a subjective assessment technique (Spielberger, 2010), has also be used as an assessment of an individual's anxiety levels in previous work (Yoon et al., 2009; Keller-Ross et al., 2014a).

### Potential Mechanisms of Fatigue Under Cognitive Stress

Cognitive stress and high cognitive demands are associated with higher rates of perceived exertion (Marcora et al., 2009), a decrease in force steadiness (Pereira et al., 2015) and enhanced EMG activity in muscles performing the physical task (Van Galen et al., 2002). Even in the absence of physical demand, cognitive stressors contribute to keeping low threshold motor units active which causes lack of muscle rest and can contribute to muscle pain (Lundberg et al., 2002). The effect of stress on force steadiness is dependent on the type of stressor and whether exercise is performed concurrently with stress or after stress. Stressors concurrently applied with exercise (like mental math) reduce force steadiness indicating poorer muscle control (Yoon et al., 2009), whereas social stressors like the Trier-Social Stress test have no impact on force variability (Shortz and Mehta, 2017). To understand the mechanisms behind fatigue under stress, the relative contributions of peripheral and central mechanisms need to be investigated. Increased sympathetic activity caused by stress induces electromyographic activity in low threshold motoneurons (Lundberg et al., 2002) and modulates muscle contractile properties and motor unit discharge rate (Roatta et al., 2008). This mechanism can potentially be responsible for increase in both peripheral or central fatigue.

Another potential cause for exacerbated neuromuscular fatigue under stress can be the effect of stress on brain activity. Cognitive stress impairs the functioning of the PFC (Arnsten, 2009), which is involved in neuromuscular performance as evidenced by several studies (Liu et al., 2003; Muthalib et al., 2013; Rhee and Mehta, 2018). According to Robertson and Marino (2016), the PFC may be involved in regulating and terminating exercise by interpreting and integrating the afferent feedback from the motor nerves and muscles through the anterior cingulate cortex, the orbitofrontal cortex, or the premotor areas. The claim of potential involvement of the PFC in regulating exercise tolerance is also supported by the fact that cognitive and emotional factors like self-motivation impact exercise performance (Barwood et al., 2015). Cognitive declines like mild cognitive impairments are also associated with low dexterity, impaired motor skills and even greater fatigue (Roalf et al., 2018; Suzumura et al., 2018; Kukla et al., 2021). Therefore, it is possible that the increased fatigability under stress is related to the temporary cognitive perturbation and stunted PFC activity caused by stress. However, no studies have investigated whether the stunted PFC activity during acute stress is directly or indirectly responsible for shorter endurance time.

Keller-Ross et al. (2014a) found that during sustained low intensity submaximal contractions, the loss in voluntary activation due to fatigue was the same with and without stress, but the time to task failure was shorter under stress. This indicates that while the relative contributions of central fatigue to neuromuscular performance were similar with and without stressor, central fatigue development was accelerated by the stressor. Furthermore, as voluntary activation was measured by the transcranial magnetic stimulation (TMS) of the motor regions, supraspinal factors were involved in central fatigue development. While the findings allude to supraspinal mechanisms being invoked in accelerated time to fatigue under stress, no studies have investigated if the same holds true for intermittent submaximal contractions that allow the progression of fatigue over a longer period of time. Also, the mechanisms behind the accelerated decline of the descending drive to the motoneurons are not established.

### Role of Individual Factors

Neuromuscular performance and fatigability are highly variable between the sexes due to physiological differences. Males exhibit higher strength than females while females have exhibited higher endurance for submaximal contractions at levels relative to the strength of various muscle groups (Hicks et al., 2001; Hunter and Enoka, 2001; Hunter, 2014). Hunter and Enoka (2001) however have attributed higher endurance for females to lower absolute strength. Hunter (2014) has attributed sex differences in fatigability to not only muscle specific factors like muscle mass, blood perfusion to muscles, muscle fiber type, and metabolism but also to central mechanisms. These central mechanisms arise due to sex differences in both structural and functional aspects of the brain physiology (Ingalhalikar et al., 2014; Duan et al., 2018; Rhee and Mehta, 2018). Studies have already shown sex differences in neuromuscular fatigue under acute cognitive stress (Yoon et al., 2009) but it is not yet identified if these differences are caused by different mechanisms of fatigue for males and females. Therefore, any investigation on the mechanisms of fatigue under acute cognitive stress also needs to study potential sex differences in these mechanisms.

Conditions like old age and obesity have also shown to exacerbate the negative influence of concurrent stressors on neuromuscular performance where older and obese individuals had even shorter endurance time under acute stress (Mehta, 2015; Shortz and Mehta, 2017). Along with reduction in endurance time, age also exacerbates decrease in force steadiness at higher mental workload and anxiety levels (Vanden Noven et al., 2014). Studies have shown that the impairment of neuromuscular performance depends on the intensity of the stressor applied and that strength is a primary predictor of neuromuscular performance under stress which is characterized by a shorter endurance time (Yoon et al., 2009; Keller-Ross et al., 2014a). This means that a greater reduction in endurance time is observed in weaker individuals under acute stress, which is not observed under low intensity stress and observed to a lesser extent in stronger individuals. Apart from strength, sex, age, and obesity, other individual specific factors like physical activity and other chronic conditions are also known to influence neuromuscular performance (Allman and Rice, 2002; Almeida et al., 2008; Keller-Ross et al., 2014b; Urquhart et al., 2019). Therefore, the interaction of these individual specific factors with stress during neuromuscular performance should also be investigated.

## Conclusion

The literature review was conducted with two aims. First, to identify and compare different methods of identifying mechanisms of fatigue. More focus was given on central and more importantly, supraspinal mechanisms of fatigue. Measurement of voluntary activation using TMS was found to be the most common way of identifying central (supraspinal) fatigue with further interpretation being provided by EMG activity of the involved muscles. However, TMS, nerve stimulation, and EMG alone cannot conclusively identify the supraspinal mechanisms of fatigue upstream of the descending drive. Current brain imaging techniques like fNIRS and ECG should be combined with the above-mentioned traditional methods to get a comprehensive understanding of the influence of cognitive stress on neuromuscular fatigue.

The second aim of the review was to identify what is currently known about the mechanisms of neuromuscular fatigue under stress. It was found that acute cognitive stress significantly reduces endurance time for low intensity contractions and this effect is dependent on contraction strength for sustained submaximal contraction and individual factures like sex, age, and obesity. Voluntary activation by cortical stimulation declines significantly faster under stress implicating supraspinal mechanisms of fatigue. Stress is also responsible for keeping low threshold motor units active even in the absence of physical effort. It was also found that stress leads to a stunted PFC activity which is also involved in neuromuscular performance and more particularly, exercise regulation. While there is substantial evidence that behaviorally, cognitive stress affects neuromuscular fatigue, little attention is paid to identify the central mechanisms of fatigue under stress. The behavior of motor cortical regions and the role of stunted PFC activity under acute stress in accelerated central fatigue has also not been investigated.

## Author Contributions

OT and RM contributed to the conception of the study and wrote sections of the manuscript. OT performed the review and drafted the first version of the manuscript. Both authors contributed to the manuscript revision, read, and approved the submitted version.

## Conflict of Interest

The authors declare that the research was conducted in the absence of any commercial or financial relationships that could be construed as a potential conflict of interest.

## Publisher's Note

All claims expressed in this article are solely those of the authors and do not necessarily represent those of their affiliated organizations, or those of the publisher, the editors and the reviewers. Any product that may be evaluated in this article, or claim that may be made by its manufacturer, is not guaranteed or endorsed by the publisher.
